# Adult Presentation of Anomalous Pulmonary Artery from the Descending Aorta: A Rare Cause of Exertional Chest Pain

**DOI:** 10.5811/cpcem.2022.2.55637

**Published:** 2022-04-25

**Authors:** Ryan Offman, Veronica Wilson, Nicholas Adams

**Affiliations:** Michigan State University College of Osteopathic Medicine, Mercy Health – Muskegon, Department of Emergency Medicine, Muskegon, Michigan

**Keywords:** anomalous pulmonary artery, pulmonary hypertension, chest pain

## Abstract

**Case Presentation:**

A 20-year-old female presented to the emergency department for evaluation of exertional, right-sided chest pain. The patient underwent a computed tomography angiogram of her chest as part of her workup, demonstrating the right lower-lobe pulmonary artery arising from the abdominal aorta.

**Discussion:**

Anomalous pulmonary arterial supply is exceedingly rare. In adult patients, it is likely to be found incidentally during workup for more common medical conditions. Symptoms may include chest pain, exertional dyspnea, or hemoptysis. The high pressure of systemic blood in a low-pressure pulmonary system can result in right heart strain, pulmonary hypertension, and high-output cardiac failure.

## CASE PRESENTATION

**Image 1:**
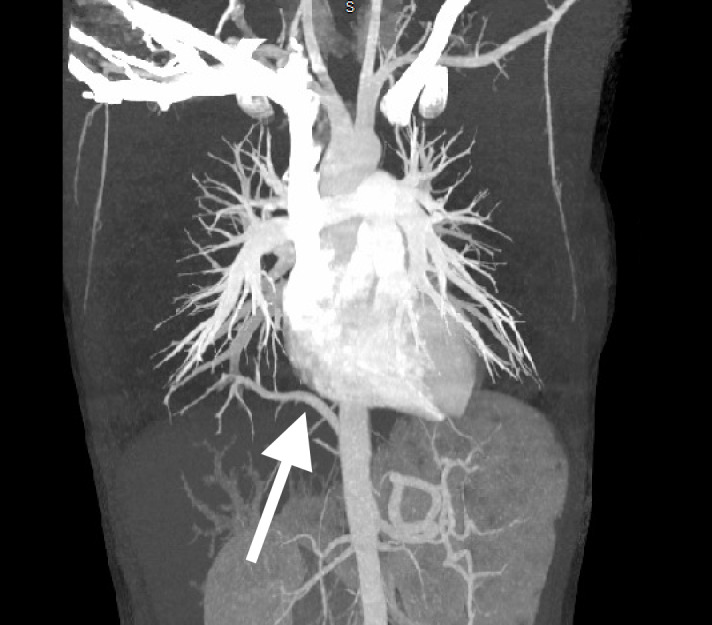
Computed tomography angiogram demonstrating the right lower lobe pulmonary artery arising from the descending aorta beyond the level of the diaphragm (arrow).

**Image 2:**
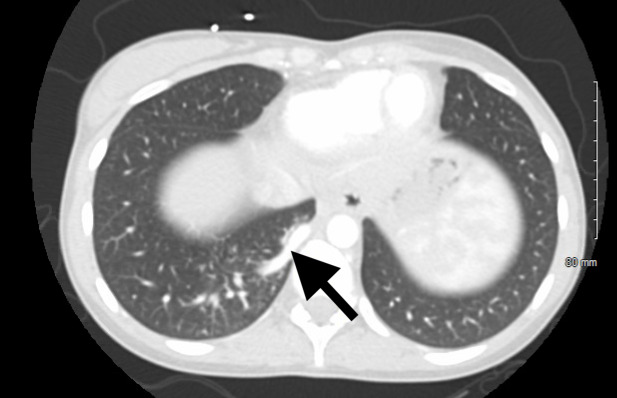
There is no evidence of pulmonary embolism or aortic dissection on the computed tomography angiogram. Arrow shows anomalous pulmonary artery originating from descending aorta.

A 20-year-old female with no previous past medical history, other than a recent evaluation for Ehlers-Danlos syndrome, presented to the emergency department for chest pain. She described exertional right-sided chest pain increasing over the previous two months. Vital signs were stable, and her physical exam was unrevealing. Electrocardiogram demonstrated poor R-wave progression in the precordial leads. Laboratory testing included an unremarkable complete blood count, comprehensive metabolic panel, and troponin. Chest radiograph was normal. Her history of possible Ehlers-Danlos syndrome prompted the emergency physician to complete a computed tomography angiogram (CTA) of the chest and abdomen for consideration of aortic dissection. The CTA revealed a pulmonary artery originating from the descending aorta above the celiac plexus, supplying the right lower lobe of the lung (Images 1 and 2). Outpatient follow-up with primary care, cardiology, and cardiothoracic surgery was ensured. Outpatient echocardiogram revealed mild tricuspid regurgitation. Definitive management of the anomalous vessel was accomplished by endovascular closure using a 10-millimeter (mm) x 7 mm Amplatzer vascular plug (Abbott Laboratories, Abbott Park, IL).

## DISCUSSION

An anomalous origin of a pulmonary artery branch is a rare congenital abnormality that comprises 0.12% of all congenital heart defects.^1^ The majority of reported cases involve left pulmonary arteries originating from the ascending aorta. Less frequently, anomalous pulmonary arteries have been described arising from the descending aorta and occasionally the celiac artery.^2^,^3^ Our patient’s specific congenital abnormality is unusual because her pulmonary artery originates from the descending aorta, supplying her right lower lobe.

Symptoms such as chest pain, hemoptysis, and exertional dyspnea may help identify patients with anomalous pulmonary vasculature. The high pressure of systemic blood in a low-pressure pulmonary system can result in right heart strain, pulmonary hypertension, and high-output cardiac failure.^4^ However, most cases are discovered in utero and are associated with other congenital anomalies. Cases that are discovered in early childhood or adolescence typically present with suboptimal weight gain, heart murmur, or abnormal chest radiograph (revealing an enlarged cardiac silhouette or persistent retrocardiac/lower lobe opacity).^3^

Understanding the significance of congenital anomalous pulmonary arteries is imperative for emergency physicians to aid in identification of this abnormality. If the patient is hemodynamically stable, patients may be followed closely as an outpatient with cardiothoracic surgery and cardiology. Surgical treatment is reserved for those with severe symptoms or those who have developed pulmonary hypertension to prevent cardiomyopathy and valvular disease. Endovascular embolization has been successful in certain cases.^5^

**CPC-EM Capsule**

What do we already know about this clinical entity?

*Anomalous pulmonary artery is a rare congenital disease that typically presents early in life with congestive heart failure*.

What is the major impact of the image(s)?

*These images are an example of a rare disease entity as a cause of chest pain and respiratory distress, which are common presenting symptoms in the emergency department*.

How might this improve emergency medicine practice?

*Although rarely discovered in adulthood, recognition of anomalous pulmonary arterial supply can lead to expedited referral and definitive management*.
